# Volunteering and resilience in wartime: evidence from two population-based studies in Israel

**DOI:** 10.1186/s12889-026-28853-5

**Published:** 2026-08-10

**Authors:** Sharon Stein Merkin, Liat Orenstein, Galia Fuchs, Dorit Efrat-Treister

**Affiliations:** 1 Research Center for Population Health, Gertner Institute for Epidemiology and Health Policy Research, Sheba Medical Center, Ramat-Gan 5262, Israel; 2 Division of Geriatrics, UCLA Geffen School of Medicine, Los Angeles, CA 90095 USA; 3 Department of Tourism and Leisure Management, Guilford Glazer Faculty of Business and Management, Ben-Gurion University of the Negev, David Ben Gurion Blvd 1, Beer-Sheva, Israel; 4 Department of Management, Guilford Glazer Faculty of Business and Management, Ben-Gurion University of the Negev, David Ben Gurion Blvd 1, Beer-Sheva, Israel

**Keywords:** Volunteering, Resilience, Social integration, Wartime trauma, Latent class analysis

## Abstract

**Background:**

Resilience is a key factor in coping with and recovering from large-scale traumatic events. Although volunteering has generally been associated with improving or maintaining resilience, evidence from wartime contexts is mixed. This study examined the association between volunteering during armed conflict and individual resilience.

**Methods:**

We used data from two population-based studies, conducted within six weeks (Study 1, *n* = 453) and 6 months after (Study 2, *n* = 1035) the October 7th, 2023 terror attacks and onset of the ensuing war in Israel. In Study 1, multivariable linear regression models were utilized to assess the association between psychological exposure to war and resilience, while accounting for potential interaction between psychological exposure to war and volunteering. In Study 2, latent class analysis was used to identify patterns of volunteering based on type, duration and frequency of activities. Multivariable linear regression models then examined associations between volunteering patterns and resilience, followed by a mediation analysis assessing the role of social integration.

**Results:**

A statistically significant interaction emerged in Study 1 indicating that the relationship between psychological war exposure and resilience was weaker when volunteering was higher. Study 2 found that the pattern characterized by high intensity volunteering at logistic distribution centers was significantly associated with higher resilience (*β* = 0.49, *p* = 0.001), with social integration mediating 31% of this effect.

**Conclusion:**

These findings show a persistent association between substantive communal volunteering and higher resilience that may buffer the negative impact of war exposure.

**Supplementary Information:**

The online version contains supplementary material available at https://doi.org/10.1186/s12889-026-28853-5.

## Introduction

### Exposure to war-related psychological trauma, and resilience

Cumulative exposure to stressors during wartime, including concomitant risks to people’s lives, health, safety, as well as loss of home and livelihood and separation from family, all amplify psychological distress [1]. Studies among civilians in conflict zones reveal heightened risks of anxiety, depression, and post-traumatic stress disorder (PTSD) [2–6], accompanied by reduced coping flexibility and optimism [7]. Evidence from the recent conflicts in Ukraine and Israel-Gaza demonstrates that individuals directly exposed to war and displacement report significantly higher levels of distress and fear [1, 8, 9] than those outside conflict zones, as well as lower resilience [10]. Meta-analytic findings confirm that depression and anxiety are significantly more prevalent during active conflicts than in post-war periods [11]. Given this high exposure to compounding stressors during war and associated deteriorations in psychological functioning, social adjustment, and subjective well-being [1], building and maintaining resilience is crucial to coping with, and ultimately, to recovering from, wartime trauma.

Resilience refers to the dynamic capacity to adapt successfully to stressors or adverse conditions [12], demonstrating individuals’ ability to recover or maintain functioning under strain [13, 14]. Indeed, high levels of perceived individual resilience have been shown to offset the disruptive psychological impact on individuals living in war zones [15, 16], while studies have shown lower coping levels related to lower levels of resilience [10]. Rather than a fixed personal trait, resilience represents a process of adjustment in the face of adversity [17, 18]. Indeed, research has shown eroding levels of resilience with exposure to war-related trauma [10, 19], as well as factors that may boost resilience in times of crisis, including social support as well as social and civic engagement, and recreational activity [20].

### Volunteering and resilience

Volunteering is broadly defined as a pro-social and altruistic form of civic engagement in which individuals contribute their time, skills, or resources to benefit others without financial compensation or legal obligation [21, 22]. Volunteering is thought to foster resilience by providing social support, increasing positive emotions, and bolstering recovery from stress by restoring depleted mental resources [23, 24]; these processes cause an upward spiral that broadens and builds resilience [25]. Extensive research highlights the positive psychosocial consequences of volunteering, including fostering emotional support, social integration, and a sense of belonging, factors that are essential for restoring mental health and adaptive coping in post-crisis environments [26, 27]. Volunteering has also been shown to enhance wellbeing through mechanisms such as increased purpose, gratitude, self-esteem, and social connectedness [17, 28]. In addition, volunteering allows individuals to observe and model others’ coping strategies, transforming helping behavior into reciprocal learning that strengthens psychological adaptation [26]. The communicative and social processes embedded in volunteering, such as mutual support, identity affirmation, and generating collective meaning, mirror the mechanisms that underlie resilience development [29, 30].

In the context of armed conflict, we propose that volunteering activity may buffer some of the negative psychological consequences by promoting and maintaining resilience. The mechanisms underlying this buffering effect are both psychological and social. First, volunteering restores self-efficacy and purpose, countering the helplessness and loss of agency commonly associated with war exposure [17]. Helping others under similar circumstances fosters perspective-taking and empathy, allowing volunteers to reinterpret their suffering as meaningful contribution rather than senseless loss [26]. Second, volunteering strengthens social ties and perceived belonging, both of which are core determinants of resilience [31, 32]. Through mutual support and collective action, individuals rebuild social capital and regain trust in their communities, resources that war often destroys [33]. Empirical evidence supports these theoretical claims with studies of post-disaster and wartime volunteering showing that participation in relief efforts enhances psychological well-being, reduces distress, and facilitates recovery [26, 34]. Among survivors of large-scale disasters, volunteering has been shown to increase hope, purpose, and perceived control, thereby strengthening resilience and accelerating psychological recovery [26]. Similarly, research on Ukrainian wartime volunteers indicates that even under extreme stress, engagement in prosocial activity enhances coping capacity and preserves emotional stability [34].

Although volunteering activity has generally been associated with improving or maintaining resilience, there is some mixed evidence about the effects of volunteering on health during wartime, with a recent study indicating that volunteering in excess can be harmful to health [35]. Moreover, there is also the possibility that volunteering efforts during wartime may be a direct reaction to fear and (depending on the nature of the volunteering) may even exacerbate that fear due to experiences of shared trauma [36]. Volunteers may also face increased feelings of moral fatigue, danger, and emotional exhaustion due to continuous exposure to distressing events and human suffering [37]. Such findings underscore that while volunteering can promote psychological wellbeing, it also carries risk of burnout and secondary traumatization during wartime.

In Israel during the recent war that began on October 7, 2023, levels of civilian volunteering were particularly high, with a recent study estimating that over 60% of the general population engaged in volunteering during first 6 months of the war [35]. The findings in that study regarding the health effects of volunteering were mixed, with some evidence that volunteering was associated with lower levels of mental health deterioration among those displaced from their homes during the war, and also indication of worsening physical health among those engaged in many types of volunteering activities [35].

### General objectives

In this study, we set out to examine the association between volunteering during wartime and levels of individual resilience utilizing two unique population-based datasets collected in Israel in the aftermath of October 7, 2023 and the ensuing war. Both sub-studies conducted a survey in two independent population-based samples after October 7th (one within six weeks of the war’s onset, and the other 6 months later), and collected information about sociodemographic factors, social wellbeing, perceptions of risk, volunteering activity and levels of resilience. In this paper, we present two parallel analyses to better understand the relationship between volunteering and resilience in times of crisis. The respective aims and hypotheses of each of these studies are described in detail below.

## Study objectives and hypotheses

### Study 1

#### Objective

Does volunteering buffer the association between exposure to war-related psychological trauma and resilience? We examined whether higher exposure to war-related psychological trauma at the start of the war was associated with lower levels of resilience, and whether volunteering moderated that association.

#### Hypothesis

Psychological exposure to war is negatively related to resilience. While exposure to war typically undermines resilience by eroding emotional, social, and personal resources, volunteering can serve as a compensatory mechanism that buffers these adverse effects. We therefore hypothesized that volunteering and psychological war exposure interact, such that the relationship between psychological war exposure and resilience is weaker with higher levels of volunteering.

#### Study 2

##### Objective

Patterns of wartime volunteering, their association with resilience and the role of social wellbeing. Building on the aims of Study 1, Study 2 aims to identify patterns of volunteering, concerning activity type and intensity (frequency and duration), to see if these were differentially associated with resilience over the first 6 months of the war. In addition, this analysis assessed the role of social wellbeing, including social support (individual support) and social integration (collective, community-based support) in explaining the link between volunteering and resilience.

##### Hypothesis

We hypothesize that low or moderately intense volunteering activity (regardless of type), but not high-intensity volunteering (or multiple types), is associated with higher levels of resilience, considering that research has shown that overexposure and exhaustion can offset the benefits of volunteering [26, 28]. Moreover, we hypothesize that social support and integration partially account for the expected positive association between volunteering and resilience.

## Methods Study 1

### Study sample

Participants included Jewish Israeli men and women from all areas of Israel during the 2023 Israel-Hamas war, that began with a large-scale Hamas attack, the most devastating to Israel since its founding [38]. Data were collected six weeks after the war began, between November 19–20, 2023 (N = 515; mean age = 43 ± 16.3 years; 50% women), via an online panel (Midgam, https://www.midgampanel.com/), yielding a representative sample of the Jewish Israeli population. This analysis includes 453 of the study participants with non-missing demographic data (detailed in Section "Measures"). Participants were asked about their volunteering activity, exposure to war, and resilience. The study was approved by the Ben-Gurion University Institutional Review Board (IRB; Reference no. DT07052020) and all participants signed informed consent forms.

### Measures

#### Volunteering

Participants were asked “How many times a week do you currently volunteer?” (1 = not at all; 2 = less than once a week; 3 = once a week; 4 = 2–3 times a week; 5 = 4–5 times a week; 6 = 6–7 times a week; 7 = several times a day) and considered as a continuous measure.

#### Psychological exposure to war

Participants were asked two questions: “I felt psychological distress following the war”; “I felt threatened by the war” (1 = not at all, 7 = very much); Cronbach’s α = 0.75. Total score was calculated based on the mean of these two items. 

#### Resilience

Resilience was based on the ten item Connor-Davidson Resilience Scale (CD-RISC) [39]. Participants were asked to indicate the extent to which they agreed with statements about their ability to adapt to change and deal with hardship, challenges and pressure, using a 7-point scale (1 = not at all, 7 = very much); Cronbach’s α = 0.94. Total score was calculated based on the mean of all ten items.

#### Sociodemographic factors

Participants reported the following demographic information: age, sex, marital status, number of children, religious level, level of income, employment status and education level (these measures are described in more detail in Table 1). These variables were obtained from the internet panel dataset when participants initially registered.

**Table 1:** Descriptive statistics of Study 1 and 2 samples

	**Study 1**		**Study 2**	
	*n* = *453*		*n* = *1035*	
	*n*	%	*n*	%
**Sociodemographic**				
**Age Groups**				
18–34	*143*	*31.6*	*345*	33.3
35–49	*142*	*31.3*	*342*	33.0
50 +	*168*	*37.1*	*348*	33.6
**Age** (mean ± SD); range	*453*	44.4 ± 16; range: 18–79	*1035*	43.3 ± 15.1; range: 18–85
**Sex**				
Male	*226*	49.9	*521*	50.3
Female	*227*	50.1	*514*	49.7
**Religiosity**				
Secular	*255*	56.3	*509*	49.2
Traditional	*88*	19.4	*288*	27.8
Religious	*69*	15.2	*134*	13.0
Ultra-Orthodox	*41*	9.1	*104*	10.0
**Marital Status**				
Married	*267*	58.9	*619*	59.8
Separated/Divorced/Widowed	*46*	10.2	*129*	12.5
Single	*140*	30.9	*287*	27.7
**Education**				
< K-12	*45*	9.8	*104*	10.1
High School diploma/matriculation	*97*	21.3	*203*	19.6
Vocational	*126*	28.7	*254*	24.5
First degree or higher	*183*	40.3	*474*	45.8
Missing	*2*	0.3		
**Household monthly income (NIS)**				
< 10,999	*189*	*41.7*	*313*	30.8
11,000–16,999	*115*	*25.4*	*300*	29.5
≥ 17,000	*101*	*22.3*	*360*	35.4
Refuses/Missing	*48*	*10.6*	*45*	4.4
**Employment**				
Unemployed	*19*	*4.2*	*132*	12.8
Employed	*389*	*85.9*	*815*	78.7
Retired	*45*	*9.9*	*88*	8.5
**No. of children** (mean ± SD); range	*453*	1.8 ± 1.8; range: 0–16	*1035*	1.4 ± 1.7; range: 0–12
**Social well-being**				
**Resilience score**^#^ (mean ± SD); range	*453*	4.70 ± 1.0; range: 0–7	*1035*	5.74 ± 1.6; range: 0–8
Volunteering activity*				
None	*246*	47.8	*387*	37.4
Few times (irregular)	*–*	–	*213*	20.6
Once a month	*89*	17.3	*52*	5.0
Several times a month	*78*	15.1	*161*	15.6
Several times a week	*86*	16.7	*114*	11.0
Every day	*16*	3.1	*108*	10.4
**War exposure**				
**Psychological exposure to war**^**¶**^ (mean ± SD); range	*453*	4.0 ± 1.69; range:1–7	*1035*	5.68 ± 2.7; range:1–7

NIS: New Israeli Shekel

^#^ Study 1: Mean of 10 items corresponding to Connor-Davidson Resilience Scale (CD-RISC) [39]; response options a 7-point scale (1 = not at all, 7 = very much)

Study 2: Sum of the 2-item abbreviated CD-RISC; a 4-point scale (0 = not at all, 4 = nearly all the time) [40]

^*^Categories were based on those used in Study 2; For comparability in this table, Study 1 categories were re-classified as follows: None (= not at all), Once a month (< once a week), Several times a month (= once a week), Several times a week (= 2–5 times a week), and Every day (6–7 times a week or several times a day). Volunteering frequency for Study 1 was included as a continuous measure, range 1–7; mean 2.17, SD 1.46

^¶^ Study 1: Mean score of two questions (“I felt psychological distress following the war”; “I felt threatened by the war” (1 = not at all, 7 = very much))

Study 2: One question (current perceived level of danger/immediate threat (0 = none, 10 = very high))

### Statistical analysis

We conducted general descriptive analyses to observe distributions of all variables of interest in the analytic sample. Multivariable linear regression models were utilized to test the association between psychological exposure to war and resilience, after adjusting for sociodemographic factors. In order to assess if volunteering modified this association, we additionally adjusted for volunteering and then considered the interaction between psychological exposure to war and volunteering. Simple slope analyses were used to interpret statistically significant interaction effects. All analyses were conducted using SPSS version 31.0.1.0. Interaction results and simple slopes were conducted using PROCESS Model 1 [41] add-on to SPSS.

## Results Study 1

Descriptive results are listed in Table 1 and indicate similar distributions to Study 2 for most of the sociodemographic factors. Nonetheless, the Study 1 sample was slightly older and reflected lower levels of socioeconomic status compared to Study 2. While volunteering activity was high in Study 1 (52.2%), it was lower than the 62.6% volunteering in Study 2, possibly due to the time periods under assessment. Indeed, during the first few weeks of the war (Study 1 period), the initial shock and trauma, coupled with frequent missile and rocket attacks as well as internal displacement from border areas may have inhibited mobility and opportunities for volunteering.

**Fig. 1:**
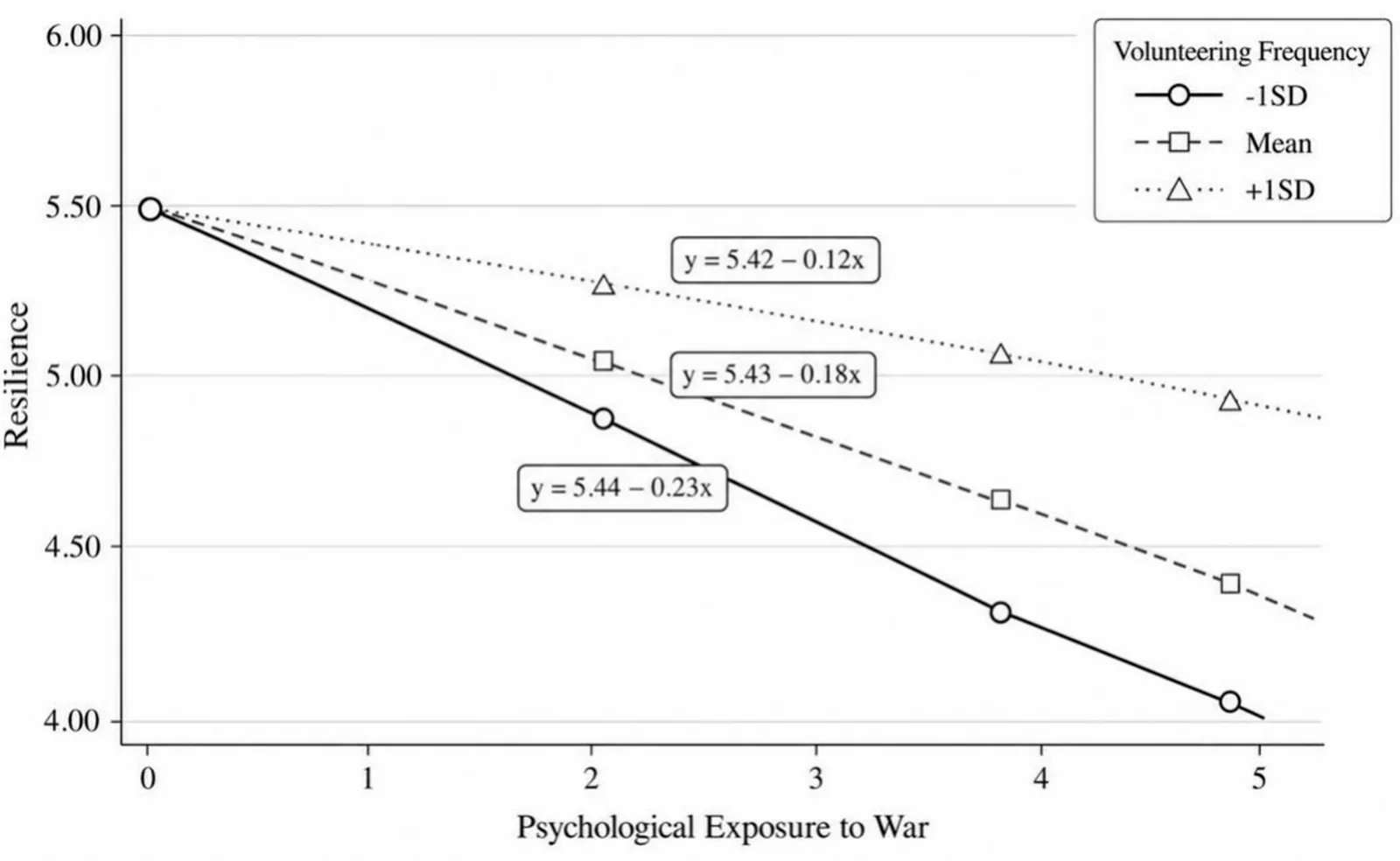
Interaction between psychological exposure to war and volunteering on resilience (Study 1). Adjusted for age, sex, marital status, religiosity, number of children in household, employment status, income, and education. To illustrate the effect, the frequency of volunteering measure was presented as Mean ± 1 SD

As expected, in the multivariable models, psychological exposure to war was negatively related to resilience (*β* = −0.17, *p* < 0.001), supporting our hypothesis, and volunteering was positively related to resilience (*β* = 0.15, *p* < 0.001; see Table 2 Model 2). There was a statistically significant interaction between volunteering and psychological war exposure on the association with resilience (*β* = 0.04, *p* = 0.03; Model 3). Simple slopes analyses illustrate these findings (Fig. 1), showing that while psychological exposure to war was significantly and negatively associated with resilience at all levels of volunteering frequency, the strength of this association weakened as volunteering frequency increased. Among participants with low volunteering frequency (1 standard deviation (SD) below the mean), the association was strongest (*β* = −0.23, p < 0.001); at mean volunteering frequency, the association was somewhat attenuated but remained statistically significant (*β* = −0.18, p < 0.001). At high volunteering frequency (1 SD above the mean), the negative association was further attenuated, though remained statistically significant (*β* = −0.12, p < 0.01; see Fig. 1).

**Table 2:** Linear regression coefficients for mean difference in resilience (Study 1)

	**Model 1**			**Model 2**			**Model 3**		
	**B**	**SE**	**p**	**B**	**SE**	**p**	**B**	**SE**	**p**
(Constant)	5.40	0.18	<.001	5.07	0.19	<.001	5.41	0.24	<.001
**Psychological exposure to war**	**−0.17**	**0.03**	** <.001**	**−0.18**	**0.03**	** <.001**	**−0.26**	**0.05**	** <.001**
**Volunteering frequency**				**0.15**	**0.03**	** <.001**	−0.01	0.08	0.89
**Volunteering frequency X Psychological exposure to war**							**0.04**	**0.02**	**0.03**
**Age group (ref: > 50)**									
35–49	0.13	0.13	0.30	0.22	0.12	0.07	0.23	0.12	0.06
18–34	0.21	0.14	0.14	**0.34**	**0.14**	**0.02**	**0.37**	**0.14**	**0.01**
**Sex (ref: Male)**									
Female	−0.13	0.10	0.18	−0.11	0.09	0.25	−0.11	0.09	0.25
**Number of children**	0.15	0.04	0.67	−0.001	0.03	0.98	−0.001	0.03	0.9
**Marital Status (ref: Married)**									
Separated/Divorced/Widowed	**0.36**	**0.16**	**0.03**	**0.35**	**0.16**	**0.03**	**0.35**	**0.16**	**0.03**
Single	−0.15	0.12	0.22	−0.18	0.12	0.14	−0.20	0.12	0.10
**Religious level (ref: Secular)**									
Traditional	−0.07	0.13	0.59	−0.06	0.12	0.61	−0.08	0.12	0.52
Religious	−0.11	0.14	0.45	−0.20	0.14	0.14	−0.19	0.14	0.17
Ultra-Orthodox	0.14	0.19	0.47	−0.02	0.19	0.90	−0.07	0.19	0.71
**Income level (ref: ≥ 17,000)**									
Lowest (< 10,999)	0.26	0.13	0.05	0.17	0.13	0.20	0.16	0.13	0.21
11,000-16,999	0.09	0.12	0.45	0.03	0.12	0.82	0.03	0.12	0.79
Missing	−0.02	0.16	0.89	−0.04	0.16	0.82	−0.04	0.16	0.79
**Education level (ref: ≥ First Degree)**									
< K-12	−0.17	0.10	0.10	−0.13	0.10	0.21	−0.12	0.10	0.24
High School diploma/matriculation	−0.28	0.17	0.11	−0.19	0.17	0.27	−019	0.17	0.27
**Employment (ref: Employed)**									
Unemployed	−0.27	0.24	0.26	−0.24	0.23	0.29	−0.23	0.23	0.32
Retired	−0.05	0.17	0.79	−0.01	0.17	0.93	0.00	0.17	1.00
**R**^**2**^** Change**	**0.125**		**<.001**	**0.042**		**<.001**	**0.009**		**0.03**

*SE* Standard Error, statistically significant (*p* < 0.05) results in **bold**. Models include unstandardized regression coefficients

## Methods Study 2

### Study sample

The study sample was based on data from the TERUMAH (Trauma, Civic Engagement, Resilience and Health) Study, a population-based survey of *n* = 1,143 Israeli adults (aged 18 and older) conducted between April 8 and May 1, 2024 (approximately six months after the October 7th events), using an online panel company (“Sekernet”). The panel company used quota sampling to ensure a generally representative distribution of age, sex and geographic region based on Israel Central Bureau of Statistics (ICBS) data. Stratified sampling was applied in this study to obtain equal representation across three age groups (18–34, 35–49, 50 +) and three main geographic regions in Israel (center, north, south) to ensure geographic representation in peripheral regions of the country that were especially impacted by the war. Full procedural details have been published [35]. The study was approved by the Sheba Medical Center IRB (Reference no. SMC-1071–24 approved on March 18, 2024) and informed consent was obtained for all participants.

To maintain comparability with Study 1, analyses were restricted to Jewish participants (*n* = 1,037). Two participants were further excluded due to missing data on war-related trauma exposure, consistent with the exclusion criteria of the original study, resulting in a final analytic sample of *n* = 1,035.

### Measures

#### Volunteering

**Fig. 2:**
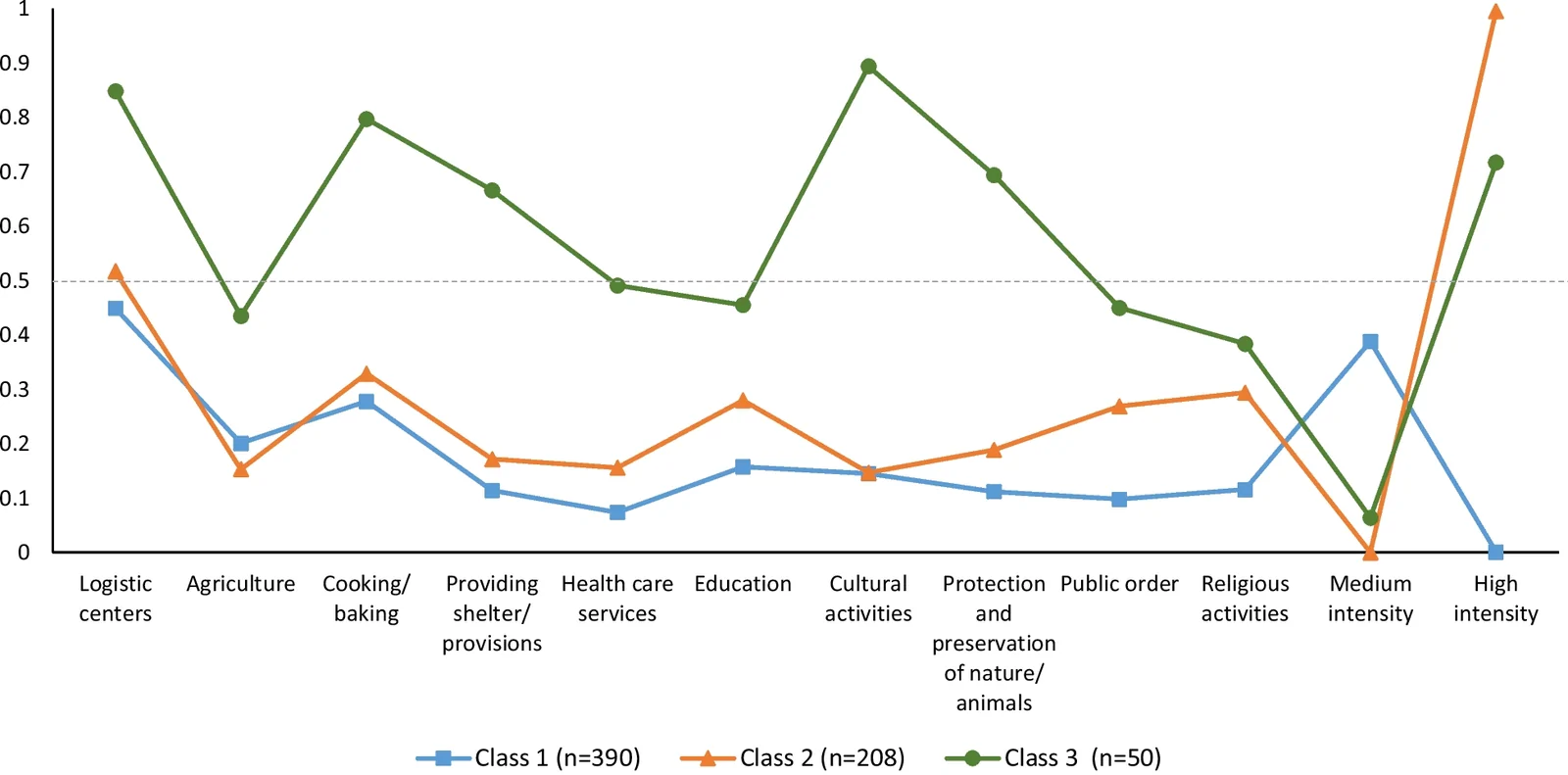
Conditional probabilities of volunteering activities by class (Study 2). Figure illustrates the characteristics of the three classes based on responses to 12 indicators of type and intensity of volunteering activity. Medium intensity refers to engagement in any volunteering activity for at least several times a month during some weeks and/or less frequently for all weeks. High intensity refers to engagement in any volunteering activity for at least several times a month during all weeks. Class 1 = ‘Low intensity’, Class 2 = ‘High intensity, logistic centers’, Class 3 = ‘High intensity, multiple types’

Participants were asked to report the type, duration and frequency of activities (for each type), and whether they had ever engaged in similar activities before October 7th. Types of activities included common types of initiatives in Israel since October 7th, mostly dedicated to helping bereaved families, families of hostages, injured civilians and soldiers, evacuated families, and families of soldiers called for reserve duty. In these analyses, we considered volunteering as reporting participation in any of these volunteering activities (list in Fig. 2), aside from financial donations. Volunteering intensity categories were based on frequency and duration during the study period (first 6 months of the war), and classified as low (< several times a month for some of the weeks), medium (≥ several times a month for some weeks and/or less frequently for all weeks), or high (≥ several times a month all weeks).

#### Psychological exposure to war

Participants were asked to indicate the level of danger/immediate threat they currently feel (Likert scale from 0- none to 10- very high level).

#### Resilience

The abbreviated CD-RISC [40] was used to measure individual resilience, based on sum of responses to 2 questions asking about the ability to adapt to change and the tendency to bounce back after hardship (Likert scale from 0- not at all to 4-nearly all the time). Cronbach’s α = 0.75.

#### Social Wellbeing

The Single Item Measure of Social Support (SIMSS) [42] was used to assess social support (defined as having at least 2 people in your life that you can count on for help in times of trouble or difficulty). For social integration, participants were asked to indicate the extent to which they agreed with the statement ‘I feel like I am part of a community’ (Likert scale from 0- strongly disagree to 4-strongly agree).

#### Sociodemographic factors

These included age (continuous and age groups: 18–34, 35–49 vs 50 +), sex, immigration status, religiosity (Ultra-Orthodox, religious, traditional, secular), marital status, number of children in household, education (highest attained degree), household monthly income (categorized into tertiles), and employment (unemployed, employed, retired).

### Statistical analysis

We conducted general descriptive analyses to observe distributions of all variables of interest in the analytic sample. We then used latent class analysis (LCA) to identify patterns of volunteering activity among participants who reported any volunteering after October 7th. LCA is a statistical modeling technique that groups individuals into distinct, unobserved subgroups (classes) based on observed indicators (categorical variables) [43]. Twelve binary variables were included, with ten reflecting specific types of volunteering activity (any vs. none) and two reflecting activity intensity (see Fig. 2). Only volunteering activities with a prevalence > 5% were included. Multicollinearity between all volunteering indicators was assessed using Cramér’s V statistic. LCA was conducted using the SAS PROC LCA procedure [44], beginning with a two-class model and systematically increased up to seven classes. For each model, 300 randomly chosen sets of initial values were used to ensure sufficient replication of the maximum likelihood [45]. Model selection was guided by goodness-of-fit indices, including log-likelihood (Log(L)), Akaike’s information criterion (AIC), Bayesian information criterion (BIC), consistent AIC (CAIC), and sample-size-adjusted BIC (aBIC), with lower values indicating better fit [43]. Diagnostic criteria included class size and entropy, with values closer to 1 indicating better class separation [43]. Finally, associations between volunteering pattern (each class membership, as well as a ‘no volunteering’ group) and demographic characteristics were examined using chi-squared or Fisher’s exact tests, as appropriate (Supplementary Table S1), with false discovery rate (FDR) adjustment applied to account for multiple testing.

Multivariable linear regression models were used to examine associations between volunteering patterns and resilience during war. First, the model was adjusted for all sociodemographic measures, psychological exposure to war, and any pre-October 7th volunteering. Next, the model was additionally adjusted for measures of social wellbeing, as these may function either as confounders or as mediators.

Finally, mediation analysis was performed to determine whether and to what extent the association between volunteering pattern and resilience could be explained by social integration. This was done using SAS PROC CAUSALMED procedure [46], which quantifies the total effect, i.e., the association between the independent (volunteering pattern) and outcome (individual resilience) variables, the direct effect (the overall effect unaffected by the mediator), and the indirect effect (the effect of the independent variable on the outcome variable attributed to the mediator).

All statistical analyses were conducted in SAS version 9.4 (SAS Institute, Inc., Cary, NC, USA).

## Results Study 2

Findings indicate that the most frequently reported activity was volunteering in logistic distribution centers (i.e., donating, packing, collecting, and/or distributing items; reported by 31.4% of participants), followed by cooking/baking (i.e., for hostage, bereaved, or displaced families, soldiers in active or reserve duty and/or their families; 21.0%), and education-related activities (e.g., in schools or nursery schools; 13.8%). Overall, 37.4% of participants did not engage in any volunteering after October 7th, whereas 14.9% reported medium-intensity involvement and 23.7% reported high-intensity involvement.

Results of the LCA for the different class models are presented in Table 3. Based on model fit statistics, specifically the BIC and cAIC, the three-class model was selected as the optimal solution. This model also demonstrated the highest entropy.

**Table 3:** Evaluating class solutions to assess volunteering clusters (Study 2)

Model fit criteria					
**Model**	**Log(L)**	**AIC**	**BIC**	**CAIC**	**ABIC**
2 class	-3969.569	1397.217	1509.064	1534.064	1429.690
3 class	-3899.696	1283.472	**1453.480**	1491.480	1332.831
4 class	-3877.668	1265.416	1493.584	1544.584	1331.661
5 class	-3853.529	1243.137	1529.466	1593.466	1326.268
6 class	-3832.051	1226.181	1570.671	1647.671	1326.198
7 class	-3812.767	1213.614	1616.264	1706.264	1330.516
**Diagnostic criteria**					
**Model**	**Smallest class count (n)**		**Smallest class size (%)**		**Entropy**
2 class	248		38		0.919
3 class	50		8		**0.933**
4 class	50		8		0.877
5 class	48		7		0.780
6 class	45		7		0.742
7 class	43		7		0.804

*N* = 648 reporting any volunteering since October 7th. Bold text indicates model met fit criteria. Log(L) = log-likelihood, *AIC* Akaike information criterion, *BIC* Bayesian information criterion, *CAIC* consistent Akaike information criterion, *ABIC* adjusted Bayesian information criterion

Figure 2 presents the conditional probabilities of each volunteering indicator by class membership, with values closer to 1 indicating a higher probability of class membership. *Class 1* (*n* = 390), labelled ‘low intensity volunteering’, was characterized by engagement in low-intensity activities without a dominant volunteering type (all conditional probabilities < 0.5). Participants assigned to this class were more likely to have a first academic degree or higher, and were less likely to be Ultra-Orthodox and to be retired (see Supplementary Table S1). *Class 2* (*n* = 208), labeled ‘high intensity, logistic centers’, was characterized by high-intensity engagement, most commonly in logistic distribution centers. Individuals in this class were more likely to be Ultra-Orthodox or religious, to have a first degree or higher, and to be retired. *Class 3* (*n* = 50), labeled ‘high intensity, multiple types’, showed high-intensity engagement, with conditional probabilities exceeding 0.5 for multiple types. All participants in this class reported volunteering prior to October 7th, were more likely to be religious, to have vocational education or no high school diploma (< K-12), and 90% were unemployed. The fourth group, those reporting no volunteering after October 7th, tended to be secular or traditional, unemployed or retired, to have an educational level lower than a first degree, and to report no prior volunteering. No significant differences in volunteering patterns were observed by age, sex or income (Supplementary Table S1).

In multivariable analysis, adjusting for sociodemographic factors, psychological exposure to war and any pre-October 7th volunteering, we found associations between patterns of wartime volunteering and resilience. Compared to no volunteering, the ‘high intensity, logistic centers’ pattern was significantly associated with higher resilience (*β* = 0.49, p = 0.001; see model 1 in Table 4). The ‘high intensity, multiple types’ pattern was also positively associated with resilience, though to a lesser extent and not statistically significant (*β* = 0.44, p = 0.07; this may be due to the much smaller sample size in this group), while ‘low intensity’ volunteering was not associated with resilience. Further adjustment to social wellbeing measures attenuated the observed associations with volunteering patterns, so that only the ‘high intensity, logistic centers’ pattern remained statistically significant, which is in line with our hypothesis. Of the two social support measures, only social integration was significantly associated with resilience (*β* = 0.40, p < 0.001; see model 2 in Table 4).

**Table 4:** Associations between volunteering profile and individual resilience 6 months after the war, *n* = 1035 (Study 2)

	**Model 1**			**Model 2**		
	**B**	**SE**	**p**	**B**	**SE**	**p**
(Constant)	**4.88**	**0.60**	** <.001**	**4.00**	**0.58**	** <.001**
**Volunteering pattern** (ref: None)						
Class 1 (low intensity)	0.09	0.12	0.47	−0.01	0.12	0.92
Class 2 (high intensity, logistic centers)	**0.49**	**0.15**	**0.001**	**0.31**	**0.15**	**0.04**
Class 3 (high intensity, multiple types)	0.44	0.25	0.07	0.17	0.24	0.47
**Psychological exposure to war**	**−0.09**	**0.02**	** <.001**	**−0.09**	**0.02**	** <.001**
**Age** (years)	**0.03**	**0.01**	**0.01**	**0.02**	**0.01**	**0.02**
**Age group** (ref: ≥ 50)						
18–34	0.43	0.31	0.16	0.37	0.30	0.21
35–49	0.22	0.20	0.26	0.26	0.19	0.17
**Sex** (ref: Male)	−0.05	0.10	0.58	−0.01	0.09	0.93
**Number of children**	0.01	0.03	0.88	0.003	0.03	0.92
**Marital Status** (ref: Married)						
Separated/Divorced/Widowed	0.30	0.16	0.05	**0.33**	**0.15**	**0.03**
Single	−0.07	0.14	0.62	−0.001	0.13	0.99
**Religious level** (ref: Secular)						
Traditional	0.06	0.11	0.57	−0.01	0.11	0.95
Religious	−0.05	0.15	0.74	−0.13	0.15	0.37
Ultra-Orthodox	−0.05	0.19	0.78	−0.32	0.18	0.08
**Income level** (ref: ≥ 17,000)						
Lowest tertile (< 10,999)	**−0.29**	**0.13**	**0.03**	−0.24	0.13	0.06
Med tertile (11,000–16,999)	−0.16	0.12	0.18	−0.14	0.12	0.22
Missing	−0.08	0.22	0.71	−0.11	0.21	0.61
**Education level** (ref: ≥ First Degree)						
< K-12	0.05	0.18	0.77	−0.04	0.17	0.82
High School diploma/matriculation	−0.16	0.14	0.25	−0.17	0.13	0.20
Vocational	0.14	0.12	0.25	0.14	0.12	0.23
**Employment** (ref: Employed)						
Unemployed	−0.14	0.15	0.36	−0.12	0.15	0.42
Retired	**−0.57**	**0.21**	**0.01**	**−0.56**	**0.20**	**0.005**
**Immigrant** (ref: No)	−0.08	0.13	0.51	−0.01	0.12	0.92
**Previously Volunteered** (ref: No)	**0.25**	**0.12**	**0.03**	**0.22**	**0.11**	**0.05**
**High Social Support (ref: No)**	-	-	-	0.17	0.10	0.10
**Social Integration**	-	-	-	**0.40**	**0.05**	** <.001**
**R**^**2**^** Change**	**0.078**		** <.001**	**0.074**		** <.001**

*SE* Standard Error, statistically significant (*p* < 0.05) results in **bold**. Models include unstandardized regression coefficients

Mediation analysis further examined the role of social integration as a possible mediator in the association between wartime volunteering and resilience. This analysis, after adjusting for all covariates, showed that the ‘high intensity, logistic centers’ volunteering pattern was associated with individual resilience, with a total effect of β = 0.45 (95% CI 0.15–0.74, *p* = 0.003; see Table 5). The indirect effect through social integration was β = 0.14 (95% CI 0.06–0.22, *p* < 0.001; percent mediated 31.0%), indicating that social integration (but not social support) statistically accounted for part of the association between volunteering activity and resilience.

**Table 5:** Mediation effect of social integration in the associations between volunteering pattern and individual resilience (Study 2)

Group	Total effect B (95% CI)	Direct effect: Path A B (95% CI)	Indirect effect: Path BC B (95% CI)	Percent mediation (%)
Model 1^**a**^	0.63 (0.38–0.89)	0.41 (0.15–0.66)	0.23 (0.14–0.31)	36.0
Model 2^b^	0.45 (0.15–0.74)	0.31 (0.02–0.59)	0.14 (0.06–0.22)	31.0


*CI* Confidence interval

^a^Adjusted for other volunteering patterns (classes 1 and 3)

^b^Additionally adjusted for age, sex, immigration status, religiosity, employment status, income, education, number of children in household, marital status, any volunteering prior to October 7th, psychological exposure to war, and social support

## Discussion

Our findings from Study 1 show that volunteering modifies the association between war-related psychological stress and lower resilience and Study 2 shows that the volunteering-resilience association depends on the nature and intensity of the volunteering activity, with the highest resilience levels associated with substantial community-based volunteering. Although longitudinal data are needed to provide further causal evidence, these findings suggest that volunteering during wartime may buffer the negative impact of exposure to war-related psychological stress on perceived resilience.

The first set of analyses (Study 1) confirms other research on the positive effects of pro-social behavior in times of crisis [47–49], although uniquely showing this effect within the first few weeks after the onset, and during, the war. Our findings show that the association between war-related stress and lower resilience was attenuated with increased frequency of volunteering. These results suggest that volunteering may soften the negative impact of war-related psychological stress on perceived resilience, although we note that the interaction effect is small and the trends are similar across all levels of resilience. Although we did not consider motivation for volunteering, other studies have shown that motivation for volunteering during wartime includes coping with stress, and that this form of engagement reduces negative feelings and strengthens social networks [34]. Thus, while our cross-sectional findings may be limited in establishing causal effects given the potential bidirectional nature of these relationships, these results nonetheless highlight the potential role of volunteering as a positive community-level method of coping during national crisis.

The second set of analyses (Study 2) provides a unique assessment of volunteering patterns that may further elucidate the positive role of volunteering during wartime as well as suggest the ways in which volunteering may improve community and individual wellbeing. Given concern about volunteer burnout and emotional exhaustion [37], we sought to assess the level and type of volunteering activity that may be most associated with resilience and thus considered type, frequency and duration of volunteering activities. Our findings suggest a patterning of volunteering that consists of two main characteristics that are most relevant to the association with resilience, including a substantial level of volunteering and volunteering in logistic centers. The first, defined in our analyses by at least several times a month of volunteering over the 6 month study period, suggests a possible threshold effect, wherein the positive impact of volunteering requires a substantial level of engagement in order to maximize benefit. This finding suggests that substantial volunteering is needed to achieve the wellbeing benefits posited by other research, including improved self-efficacy, providing meaning and purpose [26] (those measures were not included in these analyses). This was not in line with our initial hypothesis that moderate intensity volunteering would be most optimal given previous research, although that research tested a different set of outcomes and did not consider specific activity types.

It is possible that the combination of high intensity volunteering at logistic centers, the second characteristic determining this pro-resilience volunteering pattern, reflects a specifically positive volunteering pattern. Almost immediately after October 7th and the onset of the war, there were major coordinate civilian efforts centered around logistic distribution centers, providing logistics and supplies for military personnel, displaced persons, bereaved families and hostage families [50]. These logistic centers ranged from large, coordinated efforts in major cities like Tel Aviv to small family-based efforts run by private individuals or school children. In almost all cases, these centers were characterized by groups of people working alongside one another, often in a flurry of endless activity [51]. Based on our findings indicating that the pro-resilient volunteering cluster is characterized by both high levels of engagement and volunteering at logistic centers, we suggest that the communal nature of this activity was likely particularly helpful in fostering a sense of community and social integration, thereby related to higher perceived resilience. In addition, unlike activities such as volunteering in health care settings or providing shelter/provisions, which may involve direct contact with trauma survivors, engagement in logistic centers is less likely to introduce additional psychological stressors.

We further examined whether social integration might account for part of the association between volunteering and higher perceived resilience. Our results indicated that an estimated 31% of the association between volunteering and resilience was statistically explained via levels of social integration even after adjusting for sociodemographic factors, past volunteering, perceived risk and social support. This suggests a potential mediating role for social integration. However, since all variables were measured concurrently, this analysis reflects a statistical rather than causal pathway, and the directionality among these constructs cannot be inferred. Bidirectional associations and reverse causality are plausible and should be considered when interpreting these findings.

Interestingly, the volunteering pattern characterized by multiple high-intensity activity types was not significantly associated with higher resilience. These results support our hypothesis and align with earlier findings showing diminished health benefits of volunteering in too many type of activities [35]. While some negative health effects were associated with such engagement in the earlier study [35], we do not see a similar negative association with resilience in the current analysis, rather, a weak and non-significant association. The different outcomes in both sets of analyses may be the reason for the differing results, where multiple types of activities may lead to burnout and take a physical toll [52], while not associated with differences in perceived resilience.

### Policy and social implications

Overall, these findings provide evidence to support a potential social intervention that could both strengthen individual resilience and benefit society via volunteer efforts during times of armed conflict and other crises. While such an intervention has multiple merits, including encouraging community engagement and enhancing social wellbeing for volunteers, society and recipients, our study also hints at the importance of considering type of volunteering engagement to maximize benefit and minimize harm. A recent study of volunteers in combat zones in Ukraine during the current war showed high levels of emotional exhaustion, and point out that volunteers often do not have the training or tools to deal with the emotional toll of such work [37]. Our study extends this research further by expanding the type of volunteering considered in a larger sample size, and also suggests that there are diminished returns associated with engaging in too many volunteering activities or no volunteering activities. Along with earlier work indicating health risks related to engaging in several types of volunteering activities in similar circumstances [35], these findings point to the need for guidance and consideration in planning or implementing volunteer efforts among civilians during times of crisis. While the current study focused on volunteering, it is important to note that other forms of civic engagement may also promote similar benefits and should be considered in future research.

### Strengths and limitations

These two studies consider a relative large group of civilians at two different sensitive points during an ongoing armed conflict. Taken together, the positive findings with regard to volunteering provide evidence for the benefits of volunteering at the acute point immediately after the invasion and massacre and the onset of the war, as well as 6 months later. Moreover, while most other studies on volunteering did not account for various types of activities [26, 34, 37], we considered individual types along with frequency and duration. Finally, we considered adjustment for multiple factors simultaneously to tease out the independent relationship between volunteering and resilience.

There are several limitations to these analyses that should be noted. The cross-sectional nature of both studies, the first at the start of the war and the second assessed about 6 months after onset, limit our ability to infer a causal association between volunteering and resilience. We acknowledge that a bi-directional association is possible, where higher levels of resilience may increase motivation for volunteering. In order to minimize this possibility, we included a measure of previous volunteering in the Study 2 models, to account for the possibility that resilient personalities may be more likely to volunteer in general (this measure was not available in Study 1). Our studies also lacked information about motivations for volunteering that can be insightful in terms of how volunteering may function to improve wellbeing [34], as well as unmeasured pesonality characteristics that may have accounted for part of the observed associations. An additional limitation is the exclusion of minority population groups in both study samples. Given the proposed mechanism of volunteering increasing social integration, targeting groups that may be alienated from mainstream culture and society, such as minority groups or immigrants, may be of particular relevance and should be considered in future studies. Finally, Study 2 uses the abbreviated two-item CD-RISC. This scale has demonstrated robust reliability and validity, with strong correlations to the full CD‑RISC and its individual items, supporting its use as an efficient proxy for the longer scale [40]. Nonetheless, it reflects core characteristics associated with resilience rather than the dynamic processes or theoretical mechanisms underlying resilient adaptation [41].

## Conclusion

Overall, our findings suggest that volunteering offers an active pathway through which individuals may transform war-induced helplessness into collective efficacy and meaning. In re-establishing social connectedness and fostering purpose, substantive communal volunteering may buffer the negative effects of war exposure on resilience. Further longitudinal evidence is needed to confirm these suggestive findings.

## Supplementary Information

Supplementary Material 1

## Data Availability

The data obtained for this study are stored as per IRB-approved guidelines. These data can be made available to researchers upon request and per study guidelines.

## References

[1] Dembitskyi S, Sydorov M, Moskotina R, Zlobina O, Kovalska Y. Effect of war and displacement on psychological distress: a study of Ukrainian citizens. Innov Eur J Soc Sci Res. 2025;38(1):137–51. 10.1080/13511610.2025.2467836.

[2] Kurapov A, Danyliuk I, Loboda A, Kalaitzaki A, Kowatsch T, Klimash T, et al. Six months into the war: a first-wave study of stress, anxiety, and depression among in Ukraine. Front Psychiatry. 2023;14:1190465. 10.3389/fpsyt.2023.1190465. 37234208 PMC10206008

[3] Nechitailo I. Mental health of the civilians in war conditions: indicators, measurement, prospects for further research (based on the example of the Kharkiv region population during the Russia’s full-Scale invasion into Ukraine). Innov Eur J Soc Sci Res. 2025;38(1):269–79. 10.1080/13511610.2025.2464757.

[4] Mohsen F, Bakkar B, Melhem S, Aldakkak S, Mchantaf D, Marrawi M, et al. Psychological health problems among Syrians during war and the COVID-19 pandemic: national survey. BJPsych Int. 2021;18(3):E8. 10.1192/bji.2021.16. 34382955 PMC8314981

[5] Nelson B, Fernandez W, Galea S, Sisco S, Dierberg K, Gorgieva G, et al. War-related psychological sequelae among emergency department patients in the former Republic of Yugoslavia. BMC med. 2004;2(1):22. 10.1186/1741-7015-2-22. 15171785 PMC425605

[6] Fekih-Romdhane F, Helmy M, Alhuwailah A, Shuwiekh H, Naser A, Maalej E, et al. Mediating effect of depression and acute stress between exposure to Israel-Gaza war media coverage and insomnia: a multinational study from five Arab countries. BMC Public Health. 2024;24:1498. 10.1186/s12889-024-18996-8. 38835005 PMC11151504

[7] Hoppen T, Priebe S, Vetter I, Morina N. Global burden of post-traumatic stress disorder and major depression in countries affected by war between 1989 and 2019: a systematic review and meta-analysis. BMJ Glob Health. 2021;6(7):1–13. 10.1136/bmjgh-2021-006303. PMC8319986 34321235

[8] Natan M, Shapiro R, Schwartz I, Aviv R. Factors influencing nursing students’ willingness to volunteer during wartime emergencies: A cross sectional study in Israel. Nurse Educ Today. 2025;144:106458. 10.1016/j.nedt.2024.106458. 39423597

[9] Shahrabani S, Rosenboim M, Shavit T, Benzion U, Arbiv M. “Should I stay or should I go?” Risk perceptions, emotions, and the decision to stay in an attacked area. Int J Stress Manag. 2019;26(1):57.

[10] Efrat-Treister D, Fuchs G. Resilience-building leisure activities during war. Tour Recreat Res. 2025:1–13. 10.1080/02508281.2025.2559251.

[11] Lim I, Tam W, Chudzicka-Czupała A, McIntyre R, Teopiz K, Ho R, et al. Prevalence of depression, anxiety and post-traumatic stress in war-and conflict-afflicted areas: A meta-analysis. Front psychiatry. 2022;13:978703. 10.3389/fpsyt.2022.978703. 36186881 PMC9524230

[12] Bonanno G. Loss, trauma, and human resilience: have we underestimated the human capacity to thrive after extremely aversive events? Am Psychol. 2004;59(1):20. 14736317 10.1037/0003-066X.59.1.20

[13] Harris P. Another wrinkle in the debate about successful aging: the undervalued concept of resilience and the lived experience of dementia. Int J Aging Hum Dev. 2008;67(1):43–61. 10.2190/AG.67.1.c. 18630190

[14] Hochhalter A, Smith M, Ory M. Successful aging and resilience: applications for public health and health care. In: Resnick B, Gwyther L, Rob K, editors. Resilience in aging. New York: Springer; 2011. p. 15–29.

[15] Hazan-Liran B, Walter O. Coping under Crisis: The interplay between psychological capital, anxiety, and emotional eating in conflict zones. Soc Sci Humanit Open. 2025;11:101562. 10.1016/j.ssaho.2025.101562.

[16] Masten A. Ordinary magic: resilience in development. New York, NY: Guilford Press; 2014.

[17] Lee R, Heo J, Chun S, Kim J. Benefits of volunteering on resilience with aging: a case study. Ann Leis Res. 2024;27(5):703–24. 10.1080/11745398.2023.2254866.

[18] Luthar S, Cicchetti D, Becker B. The construct of resilience: a critical evaluation and guidelines for future work. Child Dev. 2000;71(3):543–62. 10.1111/1467-8624.00164. 10953923 PMC1885202

[19] Ariel D, Marciano H, Kimhi S, Eshel Y, Adini B. Individual resilience as a mediator between demographic and sociopolitical factors, community resilience and psychological distress: a five-wave study following October 7. Stress Health. 2025;41(4):e70080. 10.1002/smi.70080. 40751604 PMC12317666

[20] Fuchs G, Efrat-Treister D, Westphal M. When, where, and with whom during crisis: The effect of risk perceptions and psychological distance on travel intentions. Tour Manag. 2024;100:104809. 10.1016/j.tourman.2023.104809. 37387777 PMC10290809

[21] Anheier H, Scherer N. The Oxford Handbook of Social Movements. 2014. 10.1093/oxfordhb/9780199678402.013. Accessed 13 May 2026.

[22] Musick M, Wilson J. Volunteers: a social profile. Indiana University Press; 2007.

[23] Iwasaki Y, MacKay K, Mactavish J. Gender-based analyses of coping with stress among professional managers: leisure coping and non-leisure coping. J Leis Res. 2005;37(1):1–28. 10.1080/00222216.2005.11950038.

[24] Iwasaki Y. Counteracting stress through leisure coping: a prospective health study. Psychol Health Med. 2006;11(2):209–20. 10.1080/13548500500155941. 17129909

[25] Denovan A, Macaskill A. Building resilience to stress through leisure activities: a qualitative analysis. Ann Leis Res. 2017;20(4):446–66. 10.1080/11745398.2016.1211943.

[26] Carlile J, Mauseth K, Clark N, Cruz J, Thoburn J. Local volunteerism and resilience following large-scale disaster: outcomes for health support team volunteers in Haiti. Int J Disaster Risk Sci. 2014;5(3):206–13. 10.1007/s13753-014-0028-z.

[27] Tang F, Choi E, Morrow-Howell N. Organizational support and volunteering benefits for older adults. Gerontol. 2010;50(5):603–12. 10.1093/geront/gnq020. 20211944

[28] Carlton S, Wong J. Well-being and ill-being: Prominences and differences in the perspectives of volunteers in the immediate and longer-term aftermaths of disaster. Int J Disaster Risk Reduc. 2024;113:104837. 10.1016/j.ijdrr.2024.104837.

[29] Buzzanell P. Resilience: talking, resisting, and imagining new normalcies into being. J Commun. 2010;60(1):1–14. 10.1111/j.1460-2466.2009.01469.x.

[30] Afifi T. Individual/relational resilience. J Appl Commun Res. 2018;46(1):5–9. 10.1080/00909882.2018.1426707.

[31] Charney D. Psychobiological mechanisms of resilience and vulnerability: implications for successful adaptation to extreme stress. Am J Psychiatry. 2004;161(2):195–216. 10.1176/foc.2.3.368. 14754765

[32] Gelkopf M, Berger R, Ryan P, Cotton S. The impact of ‘“training the trainers”’ course for helping tsunami-survivor children on Sri Lankan disaster volunteer workers. Int J Stress Manag. 2008;15(2):117–35.

[33] Bar‐Tal D, Jacobson D. A psychological perspective on security. Appl Psychol. 1998;47(1):59–71. 10.1111/j.1464-0597.1998.tb00013.x.

[34] Chudzicka-Czupała A, Chiang S, Tan C, Hapon N, Żywiołek-Szeja M, Karamushka L, et al. Association between mental health, psychological characteristics, and motivational functions of volunteerism among Polish and Ukrainian volunteers during the Russo-Ukrainian War. Sci Rep. 2023;13(1):20725. 10.1038/s41598-023-47840-z. 38007575 PMC10676412

[35] Merkin S, Orenstein L. Civic engagement during crisis: does volunteering buffer the impact of trauma on worsening physical and mental health? SSM Ment Health. 2025. 10.1016/j.ssmmh.2025.100445.

[36] Baum N. Shared traumatic reality in communal disasters: toward a conceptualization. Psychother Theory Res Pract Train. 2010;47(2):249–59. 10.1037/a0019784 22402051

[37] Pidbutska N, Knysh A, Demidova Y. Psychological safety of volunteers during the war. Journal of Education Culture and Society. 2023;14(1):66–75.

[38] Lahav E, Sherman A, Shavit T. The longitudinal effect of pre-war investments in hedonic capital on wartime well-being. J Happiness Stud. 2025;26(1):11. 10.1007/s10902-024-00851-7.

[39] Campbell-Sills L, Stein MB. Psychometric analysis and refinement of the Connor–Davidson resilience scale (CD-RISC): validation of a 10-item measure of resilience. J Trauma Stress. 2007;20(6):1019–28. 10.1002/jts.20271. 18157881

[40] Vaishnavi S, Connor K, Davidson J. An abbreviated version of the Connor-Davidson Resilience Scale (CD-RISC), the CD-RISC2: psychometric properties and applications in psychopharmacological trials. Psychiatry Res. 2007;152(2–3):293–7. 10.1016/j.psychres.2007.01.006. 17459488 PMC2041449

[41] Hayes A, Rockwood N. Conditional process analysis: concepts, computation, and advances in the modeling of the contingencies of mechanisms. Am Behav Sci. 2020;64(1):19–54. 10.1177/0002764219859633.

[42] Slavin V, Creedy D, Gamble J. Single Item Measure of Social Supports: Evaluation of construct validity during pregnancy. J Affect Disord. 2020;272:91–7. 10.1016/j.jad.2020.03.109. 32379626

[43] Sinha P, Calfee C, Delucchi K. Practitioner’s guide to latent class analysis: methodological considerations and common pitfalls. Crit Care Med. 2021;49(1):e63-79. 10.1097/CCM.0000000000004710. 33165028 PMC7746621

[44] Lanza S, Dziak J, Huang L, Xu S, Collins L. PROC LCA and PROC LTA user’s guide. State College, University Park, PA: The Methodology Center, Pennsylvania State University; 2015.

[45] Berlin K, Williams N, Parra G. An introduction to latent variable mixture modeling (part 1): overview and cross-sectional latent class and latent profile analyses. J Pediatr Psychol. 2014;39(2):174–87. 10.1093/jpepsy/jst084. 24277769

[46] Inc. SI. SAS/STAT® user’s guide. Cary, NC: SAS Institute Inc; 2021.

[47] Ramkissoon H. Prosociality in times of separation and loss. Curr Opin Psychol. 2022;45:101290. 10.1016/j.copsyc.2021.11.008. 35151946

[48] Varma M, Chen D, Lin X, Aknin L, Hu X. Prosocial behavior promotes positive emotion during the COVID-19 pandemic. Emotion. 2023;23(2):538. 35298223 10.1037/emo0001077

[49] Rimé B, Páez D, Basabe N, Martínez F. Social sharing of emotion, post‐traumatic growth, and emotional climate: follow‐up of Spanish citizen’s response to the collective trauma of March 11th terrorist attacks in Madrid. Eur J Soc Psychol. 2010;40(6):1029–45. 10.1002/ejsp.700.

[50] Almog-Bar M, Bar R, Barkai R, Marmus H. Civil society engagement in Israel during the Iron Swords war: Emerging trends and preliminary insights. Jerusalem: Institute for the Study of Civil Society and Philanthropy in Israel; 2023.

[51] The Times of Israel. Movements set up to oppose government’s judicial overhaul bid metamorphose overnight to coordinate unprecedented civilian rescue and relief effort. 2023. https://www.timesofisrael.com/in-stunning-response-15000-volunteers-fill-leadership-vacuum-to-help-victims-of-hamas/. Accessed 12 May 2026.

[52] Jenkinson C, Dickens A, Jones K, Thompson-Coon J, Taylor R, Rogers M, et al. Is volunteering a public health intervention? A systematic review and meta-analysis of the health and survival of volunteers. BMC Public Health. 2013;13:773. 10.1186/1471-2458-13-773. 23968220 PMC3766013

