# Supplementary material for: Volunteering and resilience in wartime: evidence from two population-based studies in Israel
Source: BMC Public Health. 2026 Aug 10;26:2575. doi: 10.1186/s12889-026-28853-5 (PMC13560447; doi:10.1186/s12889-026-28853-5)
Supplement: Supplementary file 1 [file 12889_2026_28853_MOESM1_ESM.docx]

**Volunteering and Resilience in Wartime: Evidence from Two Population-Based Studies in Israel**

**Supplementary Table S1. Characteristics of Study 2 Sample by Volunteering Patterns**

|  | **Overall, *n=1035*** | | **Non-volunteers *n=387*** | **Class 1 (Low intensity) *n=390*** | **Class 2 (High intensity, logistic centers) *n=208*** | **Class 3 (High intensity, multiple types) *n=50*** |  |
| --- | --- | --- | --- | --- | --- | --- | --- |
|  | **N** | **%** | **%** | **%** | **%** | **%** |  |
| Sociodemographics | | | | | | | |
| Age Group |  |  |  |  |  |  |  |
| 18-34 | 345 | 33.3 | 32.0 | 33.6 | 34.1 | 38.0 |  |
| 35-49 | 342 | 33.0 | 33.1 | 35.6 | 26.4 | 40.0 |  |
| 50+ | 348 | 33.6 | 34.9 | 30.8 | 39.4 | 22.0 |  |
| Sex |  |  |  |  |  |  |  |
| Male | 521 | 50.3 | 52.5 | 45.9 | 54.3 | 52.0 |  |
| Female | 514 | 49.7 | 47.5 | 54.1 | 45.7 | 48.0 |  |
| Immigrant | 181 | 17.5 | 21.2 | 14.4 | 16.8 | 16.0 |  |
| Religiosity ^†^ |  |  |  |  |  |  |  |
| Ultra Orthodox | 104 | 10.0 | 9.6 | 7.4 | 16.3 | 8.0 |  |
| Religious | 134 | 12.9 | 9.3 | 14.1 | 16.4 | 18.0 |  |
| Traditional | 288 | 27.8 | 28.9 | 28.5 | 25.0 | 26.0 |  |
| Secular | 509 | 49.2 | 52.2 | 50.0 | 42.3 | 48.0 |  |
| Marital Status |  |  |  |  |  |  |  |
| Married | 619 | 59.8 | 63.3 | 55.6 | 60.6 | 62.0 |  |
| Separated/ Divorced/Widowed | 129 | 12.5 | 10.8 | 15.7 | 10.6 | 8.0 |  |
| Single | 287 | 27.7 | 25.8 | 28.7 | 28.8 | 30.0 |  |
| Number of Children in Household (mean ± SD) | 1035 | 1.4 ± 1.7 | 1.4 ± 1.7 | 1.3 ± 1.6 | 1.4 ± 1.7 | 1.7 ± 1.7 |  |
| Education ^†^ |  |  |  |  |  |  |  |
| <K-12 | 104 | 10.1 | 11.4 | 7.7 | 10.6 | 16.0 |  |
| High School diploma/matriculation | 203 | 19.6 | 22.0 | 19.5 | 16.3 | 16.0 |  |
| Vocational | 254 | 24.5 | 28.4 | 20.8 | 22.6 | 32.0 |  |
| First degree or higher | 474 | 45.8 | 38.2 | 52.1 | 50.5 | 36.0 |  |
| Household Monthly Income (NIS; before taxes) |  |  |  |  |  |  |  |
| < 10,999 | 313 | 30.2 | 30.5 | 31.5 | 28.8 | 24.0 |  |
| 11,000-16,999 | 300 | 29.0 | 28.2 | 27.9 | 30.8 | 36.0 |  |
| ≥17,000 | 360 | 34.8 | 33.1 | 34.9 | 38.5 | 32.0 |  |
| Refuse/Don’t know | 62 | 6.0 | 8.3 | 5.6 | 1.9 | 8.0 |  |
| Employment |  |  |  |  |  |  |  |
| Unemployed | 132 | 12.8 | 14.2 | 13.3 | 10.1 | 8.0 |  |
| Employed | 815 | 78.7 | 74.9 | 80.8 | 79.3 | 90.0 |  |
| Retired | 88 | 8.5 | 10.9 | 5.9 | 10.6 | 2.0 |  |
| *Social Wellbeing* | | | | | | |  |
| Previously Volunteered ^†^ | 532 | 51.4 | 16.0 | 62.6 | 84.6 | 100 |  |
| High Social Support | 721 | 69.7 | 68.5 | 66.9 | 76.0 | 74.0 |  |
| Social Integration (mean ± SD) ^†^ | 1035 | 2.7 ± 1.1 | 2.4 ± 1.1 | 2.7 ± 1.0 | 3.0 ± 1.0 | 3.1 ± 0.9 |  |
| Individual Resilience Score (mean ± SD) ^†^ | 1035 | 5.7 ± 1.6 | 5.5 ± 1.6 | 5.7 ± 1.5 | 6.2 ± 1.6 | 6.0 ± 1.6 |  |
|  |  |  |  |  |  |  |  |
| Psychological exposure to war ^†^ | 1035 | 5.7 ± 2.7 | 5.5 ± 2.8 | 5.8 ± 2.5 | 5.6 ± 2.7 | 6.8 ± 2.7 |  |

Class 1: Low intensity; Class 2: High intensity, logistic centers; Class 3: High intensity, multiple types; NIS: New Israeli Shekel.

^†^ P-value<0.05 for difference by volunteering profile.
